# Collision metastasis of urothelial and prostate carcinomas to the same lymph node: a case report and review of the literature

**DOI:** 10.1186/1752-1947-6-124

**Published:** 2012-05-14

**Authors:** Tapan Bhavsar, Jun Liu, Yajue Huang

**Affiliations:** 1 Department of Pathology and Laboratory Medicine, Temple University Hospital, Philadelphia, PA, 19140, USA; 2 Department of Pathology, University of Medicine and Dentistry of New Jersey/School of Osteopathic Medicine, Stratford, NJ, USA

**Keywords:** Carcinoma, collision tumor, prostate, urothelial

## Abstract

**Introduction:**

A collision tumor is the meeting and eventual intermingling of two malignant neoplasms arising from independent topographical sites. Collision metastasis of carcinomas in the lymph nodes is a rare event. A literature search revealed only three cases of such a collision metastasis of prostatic and urothelial carcinoma, and only one of those cases had used immunohistochemical stains to distinguish the two tumors.

**Case presentation:**

We encountered a case of this rare entity in an 83-year-old African-American man who presented to our facility with increasing pelvic pain after a transurethral resection of a high-grade bladder tumor and a negative metastatic computed tomography chest, abdomen and pelvic scan investigation. A radical cystoprostatectomy was subsequently performed revealing a multi-centric, high-grade, ill-defined infiltrating urothelial carcinoma infiltrating the right pericystic soft tissue. A histopathological examination of the prostate revealed a multi-centric adenocarcinoma (Gleason 4 + 4) involving two pelvic lymph nodes. Interestingly, while the right pelvic lymph node was positive for metastatic prostatic adenocarcinoma alone, immunohistochemical studies of the left pelvic lymph node revealed a dual metastatic urothelial (cytokeratin-7 and pan-cytokeratin positive, prostate-specific antigen and cytokeratin-20 negative) and prostatic (prostate-specific antigen and pan-cytokeratin positive, cytokeratin-7 and cytokeratin-20 negative) carcinoma.

**Conclusions:**

The collision of metastatic urothelial carcinoma and prostatic adenocarcinoma is unusual, and their biological behavior remains uncertain. A high index of suspicion along with thorough clinical examination and immunohistochemical stain results are an integral part of differentiating collision of urothelial carcinoma from prostate carcinoma, particularly when the two tumors are in close proximity with overlapping histological features.

## Introduction

Collision tumors demonstrating the mixing and mingling of two carcinomas from two distinct topographic origins at a metastatic site are rare entities [1]. These tumors are difficult to diagnose preoperatively, and pathological identification of the dual components is often the only way to make a correct diagnosis [2]. We report a rare case of collision tumor between urothelial and prostate metastatic cancers to the same pelvic lymph node. An 83-year-old African-American man underwent a radical cystoprostatectomy after a transurethral resection (TUR) revealed a high-grade bladder tumor. A histopathological examination of the bladder tumor confirmed a high-grade papillary urothelial carcinoma and histopathological evaluation of the prostate revealed a bilateral, multi-centric adenocarcinoma (Gleason score 4 + 4). The left pelvic lymph node revealed a focus of dual metastatic urothelial and prostatic carcinomas, confirmed by a panel of immunohistochemical stains including cytokeratin (CK)7, CK20, pan-cytokeratin (pan-CK) and prostate-specific antigen (PSA). Collision metastases of carcinomas from two separate primary lesions to the same lymph node are rare. A literature search revealed only three cases of such a synchronous metastatic collision tumor involving metastatic prostatic and urothelial carcinomas; only one of those cases used immunohistochemical stains to distinguish the two tumors. To the best of our knowledge, this is only the second case using immunohistochemical staining to definitively distinguish the metastatic urothelial cancer from the prostatic focus [1,3,4].

## Case presentation

**Figure 1:**
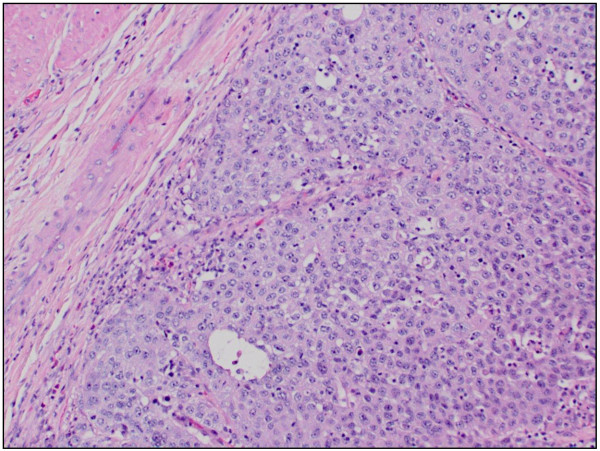
High-grade urothelial carcinoma, original tumor (hematoxylin and eosin stain, original magnification × 100).

**Figure 2:**
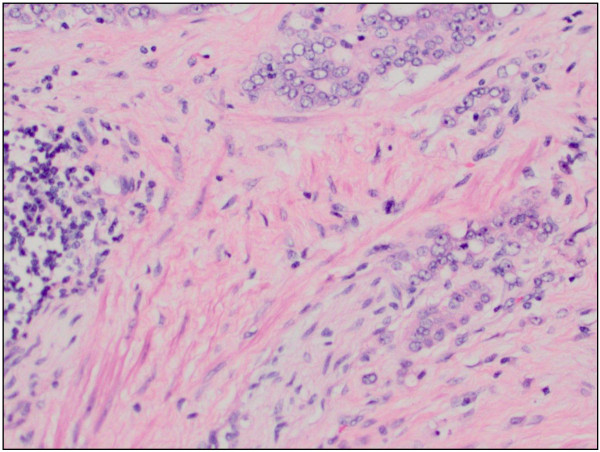
Prostate adenocarcinoma, original tumor (hematoxylin and eosin stain, original magnification × 400).

**Figure 3:**
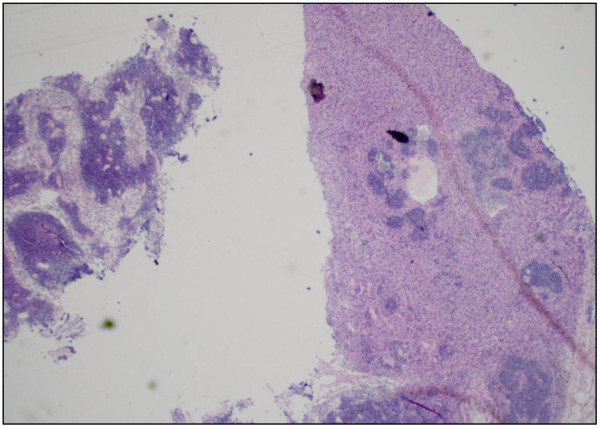
Metastatic urothelial carcinoma in the lymph node (hematoxylin and eosin stain, original magnification × 100).

**Figure 4:**
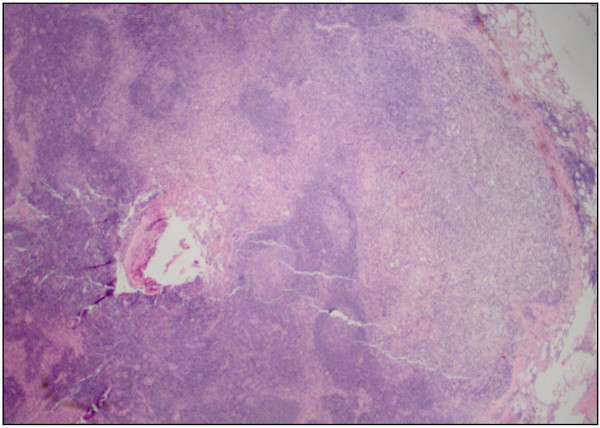
Metastatic prostatic adenocarcinoma in the lymph node (hematoxylin and eosin stain, original magnification × 100).

**Figure 5:**
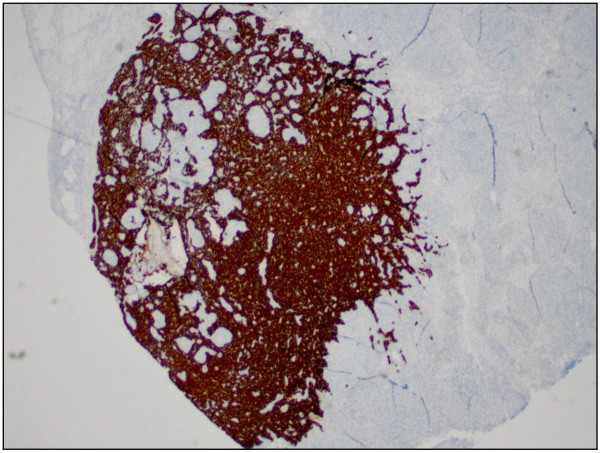
Metastatic urothelial carcinoma in the lymph node (cytokeratin stain, original magnification × 100).

**Figure 6:**
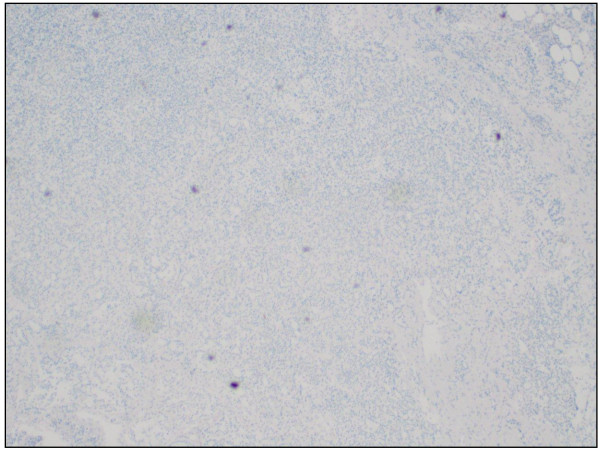
Metastatic prostatic adenocarcinoma in the lymph node (cytokeratin stain, original magnification × 100).

**Figure 7:**
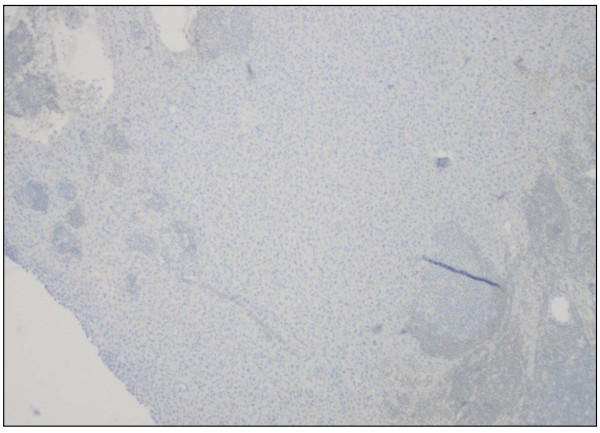
Metastatic urothelial carcinoma in the lymph node (prostate-specific antigen stain, original magnification × 100).

**Figure 8:**
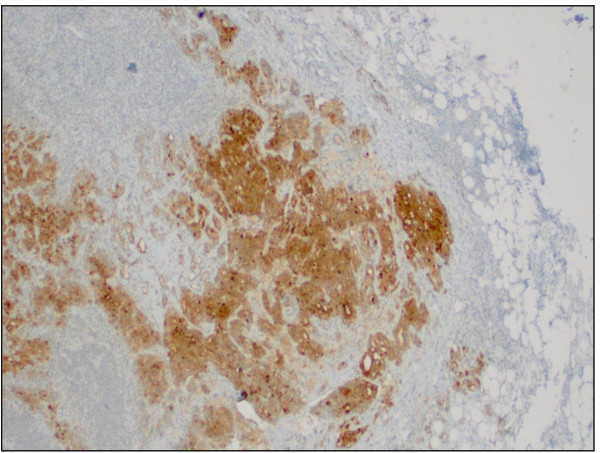
Metastatic prostatic adenocarcinoma in the lymph node (prostate-specific antigen stain, original magnification × 100).

Our patient was an 83-year-old African-American man, who was referred to our institution after originally presenting with difficult Foley placement at a local hospital. His medical history was relevant for benign prostatic hyperplasia, chronic renal insufficiency, arthritis and hypertension. His social history included tobacco use in the remote past. His vital signs and results of a review of systems were unremarkable. Results of a blood investigation showed a significant left shift with 82% segmented neutrophils. Urine analysis revealed cloudy urine, positive for leukocyte esterase, nitrites, small amount of blood and ketones. Microscopic examination of his urine showed 10 to 20 red blood cells (RBC) per high power field (HPF) and a field full of white blood cells (WBC) and bacteria. Our patient underwent cytoscopy after blood oozed out during initial catheter insertion. A complete investigation for hematuria including a computed tomography (CT) scan was performed that revealed a bladder mass. A TUR was undertaken, and histopathology confirmed the mass as being a high-grade bladder carcinoma. A follow-up metastatic investigation including a CT scan of the chest, abdomen, pelvis and bone were negative. Our patient developed increasing pelvic pain and significant hematuria. A radical cystoprostatectomy was subsequently performed revealing a multi-centric, ill-defined urothelial carcinoma (9 × 7 cm) infiltrating the right pericystic soft tissue and encompassing the right ureteral orifice. Our patient tolerated the surgical procedure well. His post-operative course was complicated by right deep venous thrombosis occluding the right common femoral vein. A histopathological examination of the bladder tumor revealed a high-grade papillary urothelial carcinoma (Figure 1) completely involving the dome and posterior wall, and partially involving the anterior and right lateral walls. The tumor extended into the perivascular soft tissue and metastatic urothelial carcinoma was identified in a left pelvic lymph node (staging: pT3; pN2; pMx). Additionally, histopathological examination of the prostate revealed a bilateral, multi-centric adenocarcinoma, Gleason 4 + 4 (Figure 2) with perineural and lymphatic/vascular invasion. Metastatic prostatic adenocarcinoma was also identified involving the left and right pelvic lymph nodes (staging: pT3a; pN1; pMx). Interestingly, the left pelvic lymph node revealed a focus of both metastatic urothelial and prostatic carcinomas (Figures 3, 4). The presence of two tumor types colliding in the same lymph node was confirmed using immunohistochemical stains, including CK7 and CK20, pan-CK and PSA. Additionally, both the primary tumors were stained with the same panel as an internal control. The focus of metastatic urothelial carcinoma was positive for CK7 (Figure 5), pan-CK, and negative for CK20, while prostatic carcinoma was negative for CK7 (Figure 6), CK20, and positive for pan-CK. In addition, the metastatic urothelial carcinoma stained negative for PSA (Figure 7), while the prostatic carcinoma was positive (Figure 8).

## Discussion

Collision tumors have been defined differently by various authors with minor variations. Meyer [5] defined this entity as ‘the meeting and eventual intermingling of two malignant neoplasms arising at independent topographical sites’. Dodge [6] suggested that, in order to accept a tumor of mixed structure as a collision tumor (that is, as the growing together of two independently arising neoplasms), there should be separate tumor areas showing two quite distinct histological patterns; furthermore, if both types of tumor metastasized, then the two types of growth should be clearly separated in the metastases also. Dodge’s definition further requires an absence of any area showing a transitional pattern that suggests a structure intermediate between the two tumor types. From Spagnolo and Heenan’s [7] point of view, collision tumors should be recognized on the basis of: (i) Two distinct, topographically separate sites of origin for the two components, and (ii) at least some separation between the two components, despite intimate mixing at the point of juxtaposition. However, in contrast to Dodge’s definition, these authors [7] allow some transitional patterns to be seen in the area of collision, and the same criteria would be applicable to metastases. This is in distinction to combination or composite tumors, which reveal divergent histologic findings and can, reveal different cellular lineages but arise from a common source [2].

Collision lymph node metastases of two carcinomas from separate sites are very rare. To the best of our knowledge, only four cases have been reported in the literature; three of which were collision metastases of prostate and bladder carcinoma [1,3,4], and one breast carcinoma metastasizing to a lymph node along with a malignant lymphoma [8].

Collision tumors, in addition to a metastatic phenomenon, such as breast carcinoma metastasizing to meningioma [9], can occur within the same organ, such as renal cell carcinoma with transitional cell carcinoma [10] or in adjacent organs, such as sigmoid adenocarcinoma with urinary bladder transitional carcinoma [11]. The incidence of this phenomenon from carcinoma arising from the genitourinary organs in comparison to other organs is relatively high due to the greater incidence of these tumors as primaries among other organs.

Several hypotheses have been suggested as mechanisms for collision tumors. The simplest is that the two primary tumors occurred in continuity by a chance accidental ‘meeting’. Two different tumors may develop contiguously because the region is altered by the same carcinogenic stimuli. Another hypothesis is that the presence of the first tumor alters the microenvironment, making the development of the second adjacent tumor more likely.

The collision of metastatic urothelial carcinoma and prostatic adenocarcinoma is unusual. The distinguishing histologic characteristics may not be clearly apparent; in fact, the two tumors may not be clearly separated in the metastases at all. This is more evident when both the tumors are poorly differentiated, equally demonstrating hyperchromasia, prominent nucleoli, atypia, and pleomorphism.

The use of immunohistochemical stains can be an integral part of differentiating high-grade urothelial carcinoma from prostate carcinoma, particularly when the two tumors are in close proximity with overlapping histologic features.

The judicious use of immunostains consisting of CK7, CK20 and PSA in differentiating prostate adenocarcinoma and bladder urothelial carcinoma has been investigated and advocated. Two studies demonstrate the usefulness of concomitant CK7 and CK20 staining to distinguish urothelial from prostate carcinoma, and merits attention. In one study, Wang *et al*. [12] stained 19 cases of urothelial carcinoma and 13 cases of prostatic carcinomas with CK7 and CK20, among multiple other tumor types. The results indicated that for urothelial carcinoma, overall 100% were CK7^+^, 89% were CK7^+^/CK20^+^, and none were CK7^-^/CK20^-^; however, for prostate carcinomas, 62% were CK7^-^/CK20^-^, and only 8% was CK7^+^[4]. On a similar note, Chu *et al*. [13] in their study staining for multiple epithelial neoplasms, demonstrated 88% of the urothelial carcinomas to be CK7^+^, 25% to be CK7^+^/CK20^+^, while 100% of the prostate carcinomas to be CK7^-^/CK20^-^. Another study by Bassily *et al*. [14] evaluated only prostate and urothelial carcinomas, staining both with CK7, CK20, and PSA. The results showed that 23 (82%) of 28 urothelial carcinomas were CK7^+^, 18 (64%) were CK20^+^ and only 6 (10%) of 59 prostate carcinomas were both CK7^+^ and CK20^+^. Even though 48 (81%) of 59 prostate carcinomas were negative for both cytokeratins, most of their urothelial tumors stained for CK7, CK20, or both. Conversely, 58 (98%) of 59 prostate carcinomas stained for PSA, but no urothelial tumors stained for PSA. The findings suggested that a combination of PSA, CK7, and CK20 is more helpful than CK7 and CK20 alone [14]. In accordance with these findings, although we suspected both urothelial carcinoma and prostate adenocarcinoma morphologically in the same lymph node, we used immunohistochemical stains to confirm and differentiate the exact metastatic foci of each tumor. The focus of metastatic urothelial carcinoma was positive for CK7 and pan-CK, and negative for PSA and CK20, while the prostatic carcinoma was positive for PSA and pan-CK and negative for CK7 and CK20.

The morphologic differentiation of metastatic urothelial from prostate carcinoma in the collision tumor is as important as the differentiation between the corresponding primary tumors, especially poorly differentiated prostate adenocarcinoma extending into the bladder neck versus high-grade urothelial carcinoma extending into the bladder neck and prostate. Since both these tumors can present with similar high-grade histologic and nuclear features, distinction by morphology alone can be difficult. In a study in 1996, Lindeman and Weidner [15] stained 29 prostate adenocarcinomas, 31 urothelial tumors and 5 poorly differentiated carcinomas of uncertain type (prostatic or urothelial origin) located at the junction of bladder neck and prostate with CK7, CK20, PSA, PAP (Prostatic Acid Phosphatase) and CEA (Carcinoembryonic Antigen). Of the 5 tumors, 3 stained for three markers including PSA, PAP and CK7. This immunologic overlap of the urothelial and prostatic tissue has been thought to be due to a common derivation from the urogenital sinus. Of course, the tailor-fit distinction into either category in these complex cases is not feasible.

Although usually considered to be merely an academic curiosity, collision tumors are clinically relevant in that the individual tumors may require different treatments. The biological behavior remains uncertain; however, most of the collision tumors are thought to carry a poor prognosis. This poor prognosis of collision tumor is dependent on the biological behavior of each original tumor or on the progress of the disease, irrespective of the collision in different nodes. In one such case, the presence and degree of differentiation of an adenocarcinoma component seemed to be more detrimental than a carcinoid component [16]. Determination of somatic genetic alterations [17] may complement the morphological and immunological criteria to determine the biclonal origin of a collision tumor.

## Conclusions

The significance of collision tumors is threefold; the diagnosis of one type of cancer does not rule out the simultaneous presence or later development of a different type of cancer, a high index of suspicion is warranted while evaluating lymph nodes in a patient diagnosed with two distinct types of cancer, and the use of immunohistochemical stains can be an integral part of differentiating all types of collision tumors from both the genitourinary tract and other organ systems.

## Consent

Written informed consent was obtained from the patient for publication of this case report and any accompanying images. A copy of the written consent is available for review by the journal’s Editor-in-Chief.

## Competing interests

The authors declare that they have no competing interests.

## Authors’ contributions

TB conceived the case report, acquired the patient data, searched the literature, and drafted the manuscript. JL performed the gross examination of the specimen, and made revisions to the manuscript. YH made critical revisions to the manuscript. All authors read and approved the final manuscript.
